# Supplementary material for: Stress combined with loss of the Candida albicans SUMO protease Ulp2 triggers selection of aneuploidy via a two-step process
Source: PLoS Genet. 2022 Dec 27;18(12):e1010576. doi: 10.1371/journal.pgen.1010576 (PMC9829183; doi:10.1371/journal.pgen.1010576)
Supplement: S1 Fig — (A) Whole genome sequence data were plotted as the log2 ratio and converted to chromosome copy number (y-axis, 1–4 copies) as a function of chromosome position (x-axis, Chr1-ChrR) using YMAP. Heterozygous (AB) regions are indicated with gray shading and homozygous regions are indicated by haplotype AA (cyan) or BB (magenta). Allele ratio changes that occur within a CNV are indicated as dark blue (AAB) or purple (ABB). Colony B and C had allele ratio colouring that was corrected using IGV and allele frequency information. (B) Serial dilution assay of ulp2Δ/Δ parental (P) and fluconazole-recovered isolates (FLC-2, FLC-3, FLC-4, FLC-1a, FLC-1b and FLC-1c) in non-selective (N/S) or media containing 128 μg/ml fluconazole (FLC). (PDF) [file pgen.1010576.s001.pdf]

Fig S1 *Rizzo et al*

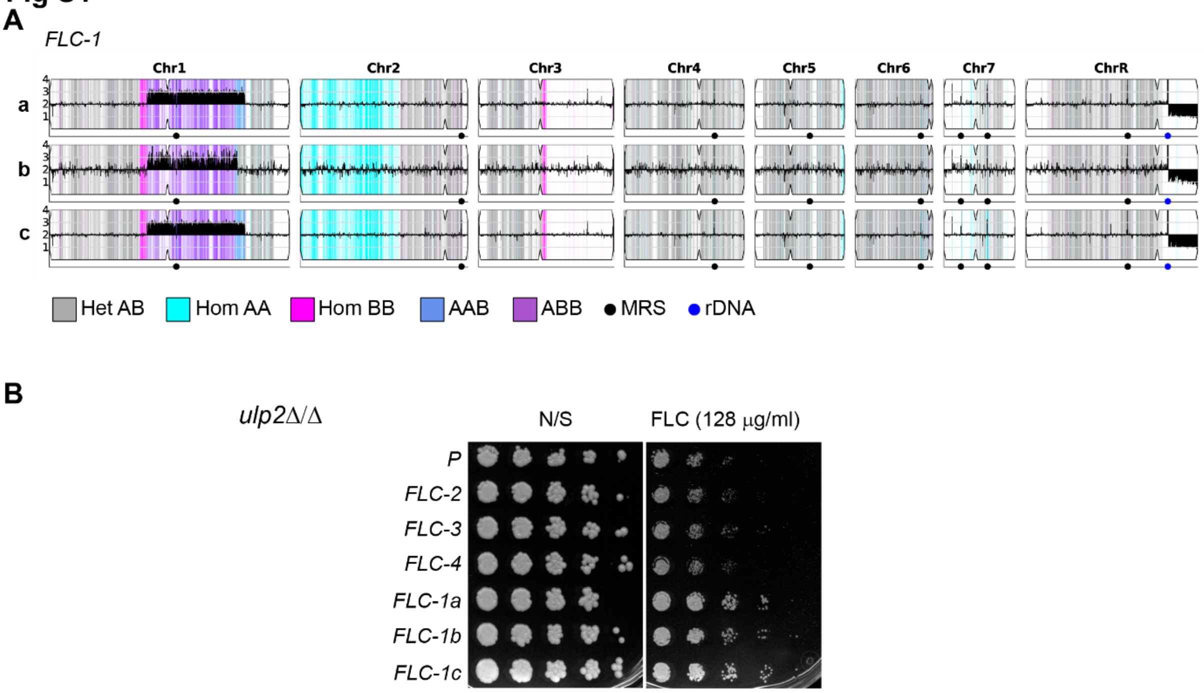

S1 Fig (A) Whole genome sequence data were plotted as the log2 ratio and converted to chromosome copy number (y-axis, 1-4 copies) as a function of chromosome position (x-axis, Chr1-ChrR) using YMAP. Heterozygous (AB) regions are indicated with gray shading and homozygous regions are indicated by haplotype AA (cyan) or BB (magenta). Allele ratio changes that occur within a CNV are indicated as dark blue (AAB) or purple (ABB). Colony B and C had allele ratio colouring that was corrected using IGV and allele frequency information. **(B)** Serial dilution assay of *ulp2*Δ/Δ parental (P) and fluconazole-recovered isolates (*FLC-2, FLC-3*, *FLC-4, FLC-1a, FLC-1b* and *FLC-1c*) in non-selective (N/S) or media containing 128 μg/ml fluconazole (FLC)
