# Supplementary material for: Stress combined with loss of the Candida albicans SUMO protease Ulp2 triggers selection of aneuploidy via a two-step process
Source: PLoS Genet. 2022 Dec 27;18(12):e1010576. doi: 10.1371/journal.pgen.1010576 (PMC9829183; doi:10.1371/journal.pgen.1010576)
Supplement: S1 Table — (DOCX) [file pgen.1010576.s002.docx]

**S1 Table:** Genetic screen top hits (score ≥2)

|  | **ORF identifier** | **Name** | **Pathway** | **MMS Score** | **UV Score** | ***S. cerevisiae* ortholog genes** |  |
| --- | --- | --- | --- | --- | --- | --- | --- |
| **1** | ORF19.3407 | *RAD18* | DNA Damage Response | 4 | 4 | *RAD18/YCR066W* |  |
| **2** | ORF19.3944 | *GRR1* | Cell Division | 4 | 4 | *GRR1/ YJR090C* |  |
| **3** | ORF19.5485 | *MEC3* | DNA Damage Response | 4 | 4 | *MEC3/ YLR288C* |  |
| **4** | ORF19.7353 | *KIP3* | Cell Division | 4 | 4 | *KIP3/ YGL216W* |  |
| **5** | ORF19.5915 | *DUR35* |  | 4 | 4 | *DUR3/YHL016C^#^* |  |
| **6** | ORF19.4350 | *PPH3* | DNA Damage Response | 4 | 0 | *PPH3/YDR075W* |  |
| **7** | ORF19.4353 | *ULP2* | Cell Division | 3 | 3 | *ULP2* |  |
| **8** | ORF19.4567 | *HQD2* |  | 3 | 3 | *N/A* |  |
| **9** | ORF19.4412 | *REV1* | DNA Damage Response/ | 3 | 3 | *REV1/ YOR346W* |  |
| **10** | ORF19.3901 |  |  | 3 | 0 | *N/A* |  |
| **11** | ORF19.2538 | *PTC2* |  | 3 | 0 | *PTC2/YER089C* |  |
| **12** | ORF19.5776 | *TOM1* | Cell Division | 2 | 4 | *TOM1/YDR457W* |  |
| **13** | ORF19.4312 | *SPT8* |  | 2 | 1 | *SPT8/YLR055C* |  |
| **14** | ORF19.4831 |  |  | 2 | 0 | *N/A* |  |
| **15** | ORF19.691 | *GPD2* |  | 2 | 0 | *GPD1* |  |
| **16** | ORF19.328 | *NPR2* |  | 1 | 3 | *NPR2/YEL062W* |  |
| **17** | ORF19.1814 | *STT4* |  | 1 | 2 | *STT4/YLR305C* |  |
| **18** | ORF19.333 | *FCY2* |  | 1 | 3 | *FCY2/YER056C* |  |
| **19** | ORF19.895 | *HOG1* | Stress Response | 1 | 2 | *HOG1/ YLR113W* |  |
| **20** | ORF19.7186 | *CLB4* | Cell Division | 1 | 2 | *CLB4/YLR210W* |  |
| **21** | ORF19.663 | *GIN4* | Cell Division | 0 | 3 | *GIN4/ YDR507C* |  |
| **22** | ORF19.8485 | *RAD32* | DNA Damage Response | 0 | 3 | *RAD30/YDR419W* |  |
| **23** | ORF19.290 | *KRE5* |  | 0 | 2 | *KRE5/ YOR336W* |  |
| **24** | ORF19.564 | *KAR3* | Cell Division | 0 | 2 | *KAR3/YPR141C* |  |
| **25** | ORF19.5662 | *PEP7* |  | 0 | 2 | *PEP7* |  |
| **26** | ORF19.6011 | *SIN3* |  | 0 | 2 | *SIN3* |  |
| **27** | ORF19.5300 | *CNE1* |  | 0 | 2 | *CNE1* |  |
| **28** | ORF19.6411 | *VAC14* |  | 0 | 2 | *VAC14* |  |
