# Supplementary material for: Stress combined with loss of the Candida albicans SUMO protease Ulp2 triggers selection of aneuploidy via a two-step process
Source: PLoS Genet. 2022 Dec 27;18(12):e1010576. doi: 10.1371/journal.pgen.1010576 (PMC9829183; doi:10.1371/journal.pgen.1010576)
Supplement: S8 Table — (DOCX) [file pgen.1010576.s009.docx]

**S8 Table: Strains used in this study**

| **Strain number** | **Name** | **Genotype** | **Source** | **SRR** |
| --- | --- | --- | --- | --- |
| AB55 | *SC5314* | WT | [1] |  |
| AB54 | *SN152*  AMS2810 | *MTL a/α ura3∆-iro1∆::imm434/URA3^+^-IRO1 his1∆/his1∆ arg4∆/arg4∆ leu2∆/leu2∆* | [2] | SRR22538153 |
| AB140 | *TetR-GFP, TetO-CEN7* | *ORF19.1963::TetR-GFP-Nat::ORF19.1963/ORF19.1963 TetO-HIS::CEN7* | [3] |  |
| AB653 | *PHO85/pho85Δ::URA3^+^* | *ura3Δ::λimm434/ura3Δimm434 his1::hisG/his1::hisG PHO85/pho85* Δ*::URA3^+^* | [4] |  |
| AB655 | *CLB4/clb4∆::URA3^+^* | *ura3Δ::λimm434/ura3Δimm434 his1::hisG/his1::hisG CLB4/clb4 Δ::URA3* | [4] |  |
| AB663 | *CRZ1/CRZ1::GFP-URA3^+^* | *ura3Δ::λimm434/ura3Δimm434 his1::hisG/his1::hisG CRZ1/CRZ1::GFP-URA3* | [4] |  |
| AB755 | *ulp1∆/∆* | *MTL a/α ura3∆-iro1∆::imm434/URA3-IRO1 his1∆/his1∆ arg4∆/arg4∆ leu2∆/leu2∆ ulp1∆::NATR/ulp1∆::ARG^+^* | This work |  |
| AB758 | *ulp2∆/∆* | *MTL a/α ura3∆-iro1∆::imm434/URA3-IRO1 his1∆/his1∆ arg4∆/arg4∆ leu2∆/leu2∆ ulp2∆::NATR/ulp2∆::ARG^+^* | This work |  |
| AB746 | *SN250* | *his1Δ/his1Δ, leu2Δ::C.dub HIS1^+^ /leu2Δ::C.maltosa LEU2^+^, arg4Δ /arg4Δ, URA3/ura3Δ::imm^434^, IRO1/iro1Δ::imm^434^* | [2,5] |  |
| AB765 | *TetR-GFP, TetO-CEN7, ulp2∆/ulp2∆* | *ORF19.1963::TetR-GFP-Nat::ORF19.1963/ORF19.1963 TetO-HIS::CEN7 ulp2 ∆::ARG^+^ /ulp2∆:: URA3^+^* | This work |  |
| AB803 | *CLB4/clb4::URA3^+^ ulp2∆/ulp2∆* | *ura3Δ::λimm434/ura3Δimm434 his1::hisG/his1::hisG CLB4/clb4∆::URA3^+^ ulp2∆::NATR / ulp2∆::HIS^+^* | This work |  |
| AB804 | *CRZ1/CRZ1::GFP-URA3^+^ ulp2∆/ulp2∆* | *ura3Δ::λimm434/ura3Δimm434 his1::hisG/his1::hisG CRZ1/CRZ1::GFP-URA3^+^* *ulp2∆::NATR* / *ulp2∆::HIS^+^* | This work |  |
| AB823 | *PHO85/pho85::URA3^+^ ulp2∆/ulp2∆* | *ura3Δ::λimm434/ura3Δimm434 his1::hisG/his1::hisG PHO85/pho85∆:: URA3^+^ ulp2∆::NATR / ulp2∆::HIS^+^* | This work |  |
| AB873 | *ulp2∆/ulp2∆ U1* AMS5495 | *MTL a/α ura3∆-iro1∆::imm434/URA3-IRO1 his1∆/his1∆ arg4∆/arg4∆ leu2∆/leu2∆ ulp2∆::NATR/ulp2∆::ARG^+^* | This work | SRR16979457 |
| AB874 | *ulp2∆/ulp2∆ U2* AMS5498 | *MTL a/α ura3∆-iro1∆::imm434/URA3-IRO1 his1∆/his1∆ arg4∆/arg4∆ leu2∆/leu2∆ ulp2∆::NATR/ulp2∆::ARG^+^* | This work | SRR16979456 |
| AB875 | *ulp2∆/ulp2∆ U3*  *AMS5499* | *MTL a/α ura3∆-iro1∆::imm434/URA3-IRO1 his1∆/his1∆ arg4∆/arg4∆ leu2∆/leu2∆ ulp2∆::NATR/ulp2∆::ARG^+^* | This work | SRR16979455 |
| AB869 | *ulp2∆/ulp2∆ FLC-2*  AMS5494 | *MTL a/α ura3∆-iro1∆::imm434/URA3-IRO1 his1∆/his1∆ arg4∆/arg4∆ leu2∆/leu2∆ ulp2∆::NATR/ulp2∆::ARG^+^* | This work | SRR16979458 |
| AB868 | *ulp2∆/ulp2∆ FLC-3*  AMS5493 | *MTL a/α ura3∆-iro1∆::imm434/URA3-IRO1 his1∆/his1∆ arg4∆/arg4∆ leu2∆/leu2∆ ulp2∆::NATR/ulp2∆::ARG^+^* | This work | SRR16979459 |
| AB870 | *ulp2∆/ulp2∆ FLC-4*  AMS5490 | *MTL a/α ura3∆-iro1∆::imm434/URA3-IRO1 his1∆/his1∆ arg4∆/arg4∆ leu2∆/leu2∆ ulp2∆::NATR/ulp2∆::ARG^+^* | This work | SRR16979460 |
| AB791 | *ulp2∆/ulp2∆ FLC-1 (T1)* | *MTL a/α ura3∆-iro1∆::imm434/URA3-IRO1 his1∆/his1∆ arg4∆/arg4∆ leu2∆/leu2∆ ulp2∆::NATR/ulp2∆::ARG^+^* | This work |  |
| AB863 | *ulp2∆/ulp2∆ FLC-1a (T2)_* AMS 5489 | *MTL a/α ura3∆-iro1∆::imm434/URA3-IRO1 his1∆/his1∆ arg4∆/arg4∆ leu2∆/leu2∆ ulp2∆::NATR/ulp2∆::ARG^+^* | This work | SRR16979461 |
| AB862 | *ulp2∆/ulp2∆ FLC-1b*  *(T2)_* AMS 5486 | *MTL a/α ura3∆-iro1∆::imm434/URA3-IRO1 his1∆/his1∆ arg4∆/arg4∆ leu2∆/leu2∆ ulp2∆::NATR/ulp2∆::ARG^+^* | This work | SRR16979462 |
| AB861 | *ulp2∆/ulp2∆ FLC-1c*  *(T2)_* AMS 5485 | *MTL a/α ura3∆-iro1∆::imm434/URA3-IRO1 his1∆/his1∆ arg4∆/arg4∆ leu2∆/leu2∆ ulp2∆::NATR/ulp2∆::ARG^+^* | This work | SRR16979463 |
| AB1136 | *ulp2∆/ulp2∆ FLC-1a*  *(R-1)* | *MTL a/α ura3∆-iro1∆::imm434/URA3-IRO1 his1∆/his1∆ arg4∆/arg4∆ leu2∆/leu2∆ ulp2∆::NATR/ulp2∆::ARG^+^* | This work |  |
| AB1137 | *ulp2∆/ulp2∆ FLC-1a*  *(R-2)* | *MTL a/α ura3∆-iro1∆::imm434/URA3-IRO1 his1∆/his1∆ arg4∆/arg4∆ leu2∆/leu2∆ ulp2∆::NATR/ulp2∆::ARG^+^* | This work |  |
| AB1138 | *ulp2∆/ulp2∆ FLC-1a*  *(R-3)* | *MTL a/α ura3∆-iro1∆::imm434/URA3-IRO1 his1∆/his1∆ arg4∆/arg4∆ leu2∆/leu2∆ ulp2∆::NATR/ulp2∆::ARG^+^* | This work |  |

**References**

1. Odds FC, Brown AJP, Gow NAR. Candida albicans genome sequence: A platform for genomics in the absence of genetics. Genome Biology. 2004;5: 1–3. doi: 10.1186/gb-2004-5-7-230

2. Noble SM, Johnson AD. Strains and Strategies for Large-Scale Gene Deletion Studies of the Diploid Human Fungal Pathogen Candida albicans Strains and Strategies for Large-Scale Gene Deletion Studies of the Diploid Human Fungal Pathogen Candida albicans. 2005; 4(2):298-309 doi: 10.1128/EC.4.2.298-309.2005

3. Burrack LS, Applen Clancey SE, Chacón JM, Gardner MK, Berman J. Monopolin recruits condensin to organize centromere DNA and repetitive DNA sequences. Mol Biol Cell. 2013;24: 2807–19. doi:10.1091/mbc.E13-05-0229

4. Forche A, Abbey D, Pisithkul T, Weinzierl MA, Ringstrom T, Bruck D, et al. Stress alters rates and types of loss of heterozygosity in Candida albicans. mBio. 2011; 2(4):e00129-11. doi:10.1128/mBio.00129-11

5. Noble SM, French S, Kohn LA, Chen V, Johnson AD. Systematic screens of a Candida albicans homozygous deletion library decouple morphogenetic switching and pathogenicity. Nat Genet. 2010;42: 590–598. doi:10.1038/ng.605
