# Supplementary material for: Stress combined with loss of the Candida albicans SUMO protease Ulp2 triggers selection of aneuploidy via a two-step process
Source: PLoS Genet. 2022 Dec 27;18(12):e1010576. doi: 10.1371/journal.pgen.1010576 (PMC9829183; doi:10.1371/journal.pgen.1010576)
Supplement: S10 Table — (DOCX) [file pgen.1010576.s011.docx]

**S10 Table: Plasmids used in this study**

| **Plasmid** | **Description** | **AB Number** | **Source** |
| --- | --- | --- | --- |
| pHA_NAT | NAT substitution cassette  HA tagging | AB17 | [1] |
| pR3Arg46spe1 | ARG substitution cassette | AB18 | [2] |
| pGEM-His1 | HIS substitution cassette | AB20 | [2] |

**References**

1. Gerami-nejad M, Forche A, Mcclellan M, Berman J. Analysis of protein function in clinical C . albicans isolates. Yeast 2012;5314. doi: 10.1002/yea.2910

2. Wilson RB, Davis D, Mitchell AP. Rapid hypothesis testing with Candida albicans through gene disruption with short homology regions. J Bacteriol. 1999;181: 1868–74. doi: 10.1128/JB.181.6.1868-1874.1999.
