# Supplementary material for: Stress combined with loss of the Candida albicans SUMO protease Ulp2 triggers selection of aneuploidy via a two-step process
Source: PLoS Genet. 2022 Dec 27;18(12):e1010576. doi: 10.1371/journal.pgen.1010576 (PMC9829183; doi:10.1371/journal.pgen.1010576)
Supplement: Attachment — Submitted filename: ReplytoReviewers_PGENETICS-D-22-01058.docx [file pgen.1010576.s012.docx]

**PGENETICS-D-22-01058: Point-by-Point Response**

**Editor's comments** The paper addresses the topic of genome stability and adaptation in the fungal pathogen Candida albicans and identified Ulp2 as having a role in maintaining genome stability. Loss of Ulp2 results in increased sensitivity to DNA damage and in the presence of fluconazole, genome rearrangements and fluconazole resistance.

*Reviewer 1* has submitted a very positive review and has some minor suggestions for improvements.

**Response:** We thank the reviewer for the positive comments and interest in our work*.*

*Reviewer 2* raises some more sub substantive comments which may be addressed through changes in emphasis and experimental revisions. The reviewer is largely unconvinced of the hypothesis that loss of Ulp2 alone is insufficient to select for dramatically different genotypes and that additional stress (here fluconazole) is required to select for genotypic variants with adaptive traits. They point out that conclusive evidence for such assertions may require much larger experiments analysing large numbers of colonies. *In the absence of such extensive analysis, the authors may wish to revise this hypothesis to accept that genomic variants can occur in Ulp2 strains (e.g. LOH in Fig 4) and that in the presence of stress variants can be selected with adaptive traits. Insistence that chromosomal variation only occurs in the presence of additional stress would require much larger experiments.*

**Response:** We thank the editor for providing insightful suggestions. We have carefully revised our hypothesis and we acknowledge throughout the manuscript (text highlighted in magenta) that; **1**. loss of *ULP2* leads to the formation of genomic variants and **2.** exposure to additional stress leads to the selection of novel karyotypes with adaptive potential.

The reviewer also suggests examining whether this segmental duplication is stable (have the authors determined whether loss of the Chr1 element is associated with reversion to susceptibility?).

**Response:** Our hypothesis is that linChr1 can be propagated during cell division (thanks to the presence of a centromere and terminal telomeres). As the editor and reviewer suggested, it should be possible to select revertant isolates that have lost this linChr1 as this extra-chromosome is predicted not to be essential. Indeed, we have now isolated 3 *FLC-1* revertants that have lost linChr1 and have a phenotype similar to the phenotype of the *ulp2*Δ/Δ strain (New Fig 6C, 6D and 6E). These data support the hypothesis that linChr1 has an adaptive value and is necessary for growth in high FLC doses.

Overall, the paper brings new data on the role of Ulp2 in Candida albicans and how genome rearrangements can result in antimicrobial resistance. The approach, using a combinations of long read, short read and karyotype data to reconstruct genomic variants is hight informative and will be of interest to a general readership.

**Response:** We thank the editor for recognising the significance of our work.

**Reviewer #1:** This article makes a substantial advance in characterising how the SUMO protease Ulp2 influences genome stability and antifungal drug resistance in the opportunistic fungal pathogen Candida albicans. The authors employed a selective deletion library containing deletion mutants of factors important for virulence and of Candida-specific genes to screen for determinants of genome stability by testing for hypersensitity to UV radiation and the alkylating agent MMS. The screen identified 28 candidates, several were conserved factors involved in cell cycle regulation, DNA damage repair, chromosome segregation, or stress response; these are obvious hits and demonstrate that the setup of the screen is working. More importantly, there were 3 candidates with no obvious homology in the main yeast models (Saccharomyces cerevisiae and Schizosaccharomyces pombe). One of the best hits (high sensitivity to UV and MMS) was ULP2 which codes for a SUMO protease. SUMOylation is a post-translational modification which has been reported to be involved in response to environmental changes.
Candida albicans contains three ULP genes (ULP1-3). The encoded proteins are poorly conserved in S. cerevisiae with the exception for their catalytic motifs. C. albicans ULP3 is essential. Hence, the authors made independent deletions of ULP1 and ULP2 (it is good practice to not reuse strains from the deletion library) for further characterisation. As corroboration of the screen the ulp2 mutant showed reduced fitness and hypersensitivity to UV, MMS, hydroxyurea, and hydrogen peroxide. The ulp1 mutant was indiscernible from wildtype, and its characterisation was not further pursued.
Using genetic and cytological marker-loss assays the authors established that the ulp2 mutant displayed increased genome instability. 3 randomly selected ulp2 mutant colonies were whole-genome sequenced, and signs of Loss-of-Heterozygosity (LoH) were detected. However, since no gross chromosome rearrangements were observed in these strains, the authors addressed the possibility that loss of ULP2 in combination with stress would trigger larger genome reorganisations. This was tested using the antifungal fluconazole (FLC). Indeed, colonies adapted to high drug concentrations arose more readily in the ulp2 mutant than in wildtype. 4 FLC-adapted colonies were then picked at random and whole-genome sequenced using an Illumina platform. To resolve potential copy number variations and chromosome rearrangements better, one of the FLC-adapted isolates FLC-1 was also whole-genome sequenced using Oxford Nanopore technology. This enabled the dissection of the chromosome aberrancies in FLC-1. The authors then suggest a two-step process to explain the genome reorganisations they observed; this is perfectly sensible.
The data is of high quality and supports the authors' conclusions, I have only a few minor issues I have detected.

**Response:** We thank the reviewer for the positive comments and for recognising the quality and importance of our work

Please ensure that your BioProjects are public, currently they cannot be accessed.

**Response:** We will release the BioProjects to the public immediately upon acceptance of the manuscript.

Specific Comments:

- L.150: please spell out genus names of the two model yeasts, it's their first mention.

**Response:** Thanks for pointing out this omission; this has now been corrected.

- L.163: surely, this should refer to Fig 1C.

**Response:** We thank the reviewer for catching this error; this has been corrected

- L.184-198: Can you exclude that the function of Ulp1 is not redundant with one, or both, of the other Ulps? Would it be worthwhile making at least the ulp1 ulp2 double mutant to check?

**Response:** The reviewer is correct: we cannot exclude this possibility. We now acknowledge this scenario in the text " Although *ULP-1, ULP-2* and *ULP-3* may have some partially redundant functions, our results suggest that *ULP-1* does not play a major role in genotoxic stress response*".* Our opinion is that generating and analysing a *ulp1ulp2* double mutant is outside the scope of this manuscript as we plan to characterise *ULP-1* in a different study fully.

- L.220: Revise "included including".

**Response:** We thank the reviewer for catching this error; this has been corrected

- L.289: "detected a linChr1".

**Response:** This has been corrected

- L.296: space missing between "Chr1 and"

**Response:** This has been corrected

- L.353: there's a single floating quotation mark.

**Response:** This has been corrected

- L.424, L.428: It's TRIS-HCl not HCL

**Response:** This has been corrected

- Refs. 7, 10, 16, 19, 20, 21, 24, 25, 29, 30, 31, 44, 52, 61, 69, 70, 73, 74, 79, 80, 87, 90, 91, 95, 96, 97, 102, 105, 108, 110, 116 have issues (missing volume numbers, incomplete page range, missing article ID, page count instead of article, and/or obviously wrong weblink)

**Response:** We apologise for the formatting mistakes generated by our Reference Manager Software. We have now fixed all the references

**Reviewer #2**: In this paper, the authors delete Candida albicans ULP2 encoding SUMO deconjugation enzyme and analyse the consequences of loss of SUMOylation, including genome stability and its role in adaptation to antifungal fluconazole. The authors show that loss of ULP2 causes chromosome instability, which, when combined with drug selective pressure, can lead to aneuploidy that, possibly, confers drug resistance and rescues some other phenotypic consequences of ULP2 loss.

Major points:
It has been previously established that the lack of ULP2 in Saccharomyces cerevisiae is associated with slow growth, abnormal spindles, irregularly shaped colonies, higher rates of chromosomal loss, and sensitivity to UV, gamma rays, heat, MMS, and hydroxyurea. This study, thus, merely confirms S. cerevisiae ULP2 properties in the opportunistic pathogen C. albicans.Lines 239 to 241, the authors say that no whole-chromosome aneuploidies were found in ulp2Δ/Δ strain. However, in lines 243 to 244 (and Fig. 4C), they report chromosome 1 missegregation followed by reduplication of the remaining homologue in strain U1. Chromosome instability/aneuploidy in strains lacking ULP2 has to be addressed with a larger number of clones, not just three of them.
Lines 247 to 249 – The hypothesis that the stress associated with loss of ULP2 is insufficient to select for dramatically different genotypes is unsubstantiated. Authors need to address more thoroughly the question whether the lack of ULP2 alone triggers chromosome aneuploidies in C. albicans similarly to S. cerevisiae. Please, do CHEF and WGS with large number of both independent ulp2Δ/Δ clones and independent FLC clones. Present WGS profiles of all clones in the same figure.
Adaptation to fluconazole needs a more systematic study. Please, obtain more clones in independent experiments, conduct a standard broth microdilution assay to determine the minimum inhibitory concentration (MIC) in FLC1, 2, 3, 4, etc clones. Visualise segmental aneuploidies with CHEF.

**Response:** We respectfully disagree with the reviewer's general opinion of our study as we believe that our study is novel and will interest the scientific community. However, we acknowledge that the reviewer has a point and that our data suggest that: 1) loss of *ULP-2* leads to genome variation and 2) adaptive karyotypes are selected when *ulp2*Δ/Δ cells are challenged with additional stress. As suggested by the editor, we have now carefully revised our text to reflect this point. In our opinion, the experimental approach suggested by the reviewers is outside this study's scope and too expensive for our resources.

To address whether the segmental duplication in FLC1 causes fluconazole resistance, the authors have to prepare independent phenotypic revertants to fluconazole sensitivity and to determine whether the segmental duplication is lost.

**Response:** Thank you for this suggestion; we have now isolated 3 *FLC-1* revertants. Diagnostic PCR analysis demonstrates that the 3 revertants have lost linCrh1 and have a phenotype similar to *ulp2*Δ/Δ strain (New Fig 6C, 6D and 6E). These findings support the idea that linChr1 has an adaptive value that is required for growth in high FLC doses.

Overall, while adaptation to fluconazole due to segmental aneuploidies needs more studies, a portion of this work characterising the properties of C. albicans ULP2 could be published in a more specialised yeast journal.

Minor points:
Line 251 – What was the reason for using a high concentration, 128 μg/ml, of fluconazole?

**Response:** This concentration was chosen because it is a high concentration (~ 1000 fold above susceptibility breakpoint) that is likely to be selective

Numerous typos have to be checked through the manuscript

**Response:** We have now carefully proof-read the manuscript
